# Cell-Free DNA and Mitochondria in Parkinson’s Disease

**DOI:** 10.3390/ijms262311615

**Published:** 2025-11-30

**Authors:** Małgorzata Wojtkowska, Franciszek Ambrosius

**Affiliations:** 1 Institute of Molecular Biology and Biotechnology, Faculty of Biology, Adam Mickiewicz University, 61-614 Poznan, Poland; 2 Department of Neurochemistry and Neuropathology, University of Medical Sciences, 61-710 Poznan, Poland

**Keywords:** cell-free DNA (cfDNA), cell-free mitochondrial DNA (cf-mtDNA), cell-free nuclear DNA (cf-ntDNA), serum, plasma, cerebrospinal fluid, mitochondria, mitophagy, Parkinson’s disease, neuroinflammation

## Abstract

Parkinson’s disease (PD) is a progressive neurodegenerative disorder marked by the gradual and irreversible loss of neurons, especially within the substantia nigra region of the midbrain. Early and accurate diagnosis remains a significant challenge in both research and clinical practice. This difficulty is further compounded by the substantial clinical and molecular heterogeneity of PD, emphasizing the urgent need for reliable biomarkers to enhance diagnostic precision and guide therapeutic strategies. One promising candidate biomarker is cell-free DNA (cfDNA), comprising short DNA fragments composed of mitochondrial (cf-mtDNA) and nucleus-derived (cf-ntDNA) DNA. cfDNA is released into body fluids through physiological or pathological processes such as apoptosis, necrosis, NETosis, or active secretion. The presence of cfDNA in human biological fluids has been utilized for years in oncology and prenatal medicine and, more recently, it has gained attention as a non-invasive diagnostic tool in the context of neurodegenerative diseases such as PD. This review article aims to provide a comprehensive overview of the current knowledge on the origin of cfDNA, highlighting the roles of the mitochondria and cf-mtDNA in PD, mitochondria quality control, and neuroinflammation in cfDNA biogenesis. The review collates available research on cfDNA types in human serum, plasma, and CSF, sequence analysis, and its potential application as a biomarker in the diagnosis and monitoring of PD, contributing to the ongoing search for non-invasive biomarkers of neurodegenerative diseases.

## 1. Parkinson’s Disease

Parkinson’s disease (PD) is the second most prevalent neurodegenerative disorder, primarily marked by the degeneration of dopaminergic neurons in the substantia nigra (SN) and the presence of intracellular protein aggregates known as Lewy bodies (LBs).

There are two known PD variants: idiopathic or sporadic and rare familial PD. The most common is idiopathic PD, defined by the late-onset of genetic factors (e.g., mutations in leucine-rich repeat kinase 2 (LRRK2) or glucosylceramidase-β) and environmental factors like pesticide exposure, prior head injury, rural living, and intensive use of β-blockers [1]. Early-onset PD, in which subjects present with PD between the ages of 21 and 50, is often associated with familial inheritance caused by gene mutations in 18 specific chromosomal regions/PD-related loci (PARK1–18), such as the SNCA gene (α-synuclein; PARK1 and PARK4), Parkin (ubiquitin protein ligase; PARK2), DJ-1 (PARK7), or LRRK2 (PARK8) [2,3] (Table 1). It is thought that, in most populations, 3–5% of PD cases are monogenic, in most cases with a fast regressive subtype.

The pathophysiology of Parkinson’s disease appears to result from the complex interplay of aberrant α-synuclein aggregation and synaptic transport issues, with mitochondria playing a leading role in its dysfunction [4]. There is currently no effective cure for PD due to the incomplete understanding of its mechanisms and, in particular, the lack of reliable early diagnostic and therapeutic targets.

Current diagnosis primarily depends on clinical assessments, imaging techniques, and biochemical biomarkers [5]. Thus, in cases of early onset and fast progress subtypes, proper diagnosis is still nearly impossible [6]. Therefore, the identification of disease-specific and non-invasive biomarkers in body fluids could greatly enhance the ability to track PD progression, including during the drug treatment process.

**Table 1:** Genes and their products related to the familiar form of Parkinson’s disease.

PARK	Gene	Protein	Mitochondrial Function
PARK1 and PARK4	*SNCA*	Alpha synuclein (Syn)	membrane curvature and synaptic vesicle formation [7,8,9] autophagy [10,11] mitophagy [12] interacts with Complex I and impairs its function [13]
PARK2	*PRKN*	Parkin (E3 ubiquitin ligase)	mitophagy [14]
PARK6	*PINK1*	PTEN-induced putative kinase 1 (mitochondrial serine/threonine protein kinase)	mitophagy [15] mitochondria fission [16]
PARK7	*DJ-1*	Protein/nucleic acid deglycase	mitophagy late stage (recruits autophagy receptors) exerts an antioxidative stress function through scavenging reactive oxygen species, regulation of transcription and signal transduction pathways, and acting as a molecular chaperone and enzyme [17]
PARK8	*LRRK2*	Leucine-rich repeat kinase 2	increased kinase activity and altered GTPase activity, respectively [18] vesicular trafficking [19] endosomal transport [20,21], cytoskeleton dynamics and neurite growth [22,23,24,25] mitochondrial morphology [26] mitochondrial calcium regulation [27]
PARK15	*FBXO7*	F-box protein 7	Mitophagy (Parkin recruitment)
PARK22	*CHCHD2*	Coiled-coil-helix-coiled coil-helix domain 2	involved in oxidative phosphorylation or in maintenance of crista morphology [28] correlated with causes chronic oxidative stress and age-dependent neurodegeneration [29]
PARK23	*VPS13C*	Vacuolar protein sorting-associated protein 13C	phospholipid transporter localizes to the contact sites between the endoplasmic reticulum (ER) and late endosome mediate endoplasmic reticulum-phagy at late endosomes [30]

## 2. Cell-Free DNA Origins

Circulating cell-free DNA (cfDNA), found in various body fluids, has emerged as a non-invasive biomarker with growing potential for various clinical applications, particularly in the context of liquid biopsy, mostly in cancer disease and pregnancy [31,32,33]. Despite ongoing research, the biological characteristics of cfDNA remain incompletely understood, especially regarding its origin, the distribution of fragment lengths and their possible function, and the role of the mitochondria in its biogenesis. Cell-free nuclear DNA (cf-ntDNA) and mitochondrial DNA (cf-mtDNA) have been distinguished, but numerous conflicting reports exist, likely due to the heterogeneous origins of cfDNA and the diverse cellular mechanisms involved in its release [34,35].

In response to cellular stress, tissue damage, or infection, various forms of extracellular DNA can be detected as cfDNA in human fluids. Under normal physiological conditions, cfDNA levels in human blood remain very low (typically in the range 1–50 ng/mL), primarily arising due to degradation by enzymes such as DNase1 and DNase1-like 3 [36]. According to studies by Moss et al. on normal human plasma [37], cfDNA originates from granulocytes (32%), erythrocyte progenitors (30%), lymphocytes (12%), monocytes (11%), vascular endothelial cells (9%), and hepatocytes (1%). Interestingly, small fragments of DNA freely circulate in the peripheral blood of both healthy and cancer-diseased individuals. Since cfDNA has a short half-life that can vary from 4 min to 2 h, it suggests itself to applications monitoring the progress of ongoing therapy.

There are different cell death mechanisms lead to cfDNA release that can be grouped into two main categories of cfDNA origin: passive release, primarily associated with cell death, and active release from living cells, such as through exocytosis.

### 2.1. Apoptosis

**Figure 1:**
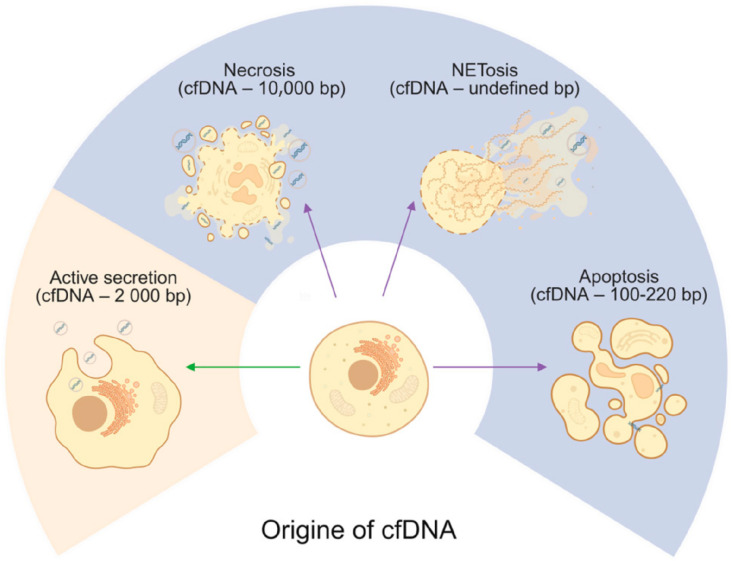
**Origins of cell-free DNA**. Cells release cfDNA through apoptosis, necrosis, NETosis, and active secretion with estimated length of the cfDNA fragments. Green arrow indicate active release from living cells, whereas violet arrows indicate passive release, primarily associated with different cell death. Created in BioRender. Ambrosius, F. (2025) https://biorender.com/4xl13l2 (accessed on 23 November 2025).

Apoptosis, or programmed cell death, is widely recognized as a major source of cell-free DNA released from both healthy and diseased tissues [38]. This tightly regulated process can be triggered by various physiological and pathological stimuli, including hormonal changes, oxidative stress, and DNA damage. It involves a cascade of molecular events mediated by caspases, leading to characteristic cellular alterations such as increased membrane permeability, cell shrinkage, chromatin condensation, and DNA fragmentation, ultimately resulting in the formation of apoptotic bodies [39]. Impaired apoptotic cell clearance, in which dead cells are not efficiently removed from the body, can lead to various human pathologies with elevated cfDNA levels [40]. CfDNA produced through apoptosis are extracellular fragments of double-stranded DNA (dsDNA) between 120 and 220 bp long, centered around 167 bp, that reflect the nucleosome pattern [41] (Figure 1). In their 2025 study, Patil et al. analyzed serum-derived cell-free DNA (cfDNA) from newly diagnosed Parkinson’s disease patients and reported discrete cfDNA length distributions, characterized by differential abundances of nucleosome fragments of approximately 150 bp [42].

### 2.2. Necrosis

Necrosis occurs when cell death is not programmed and is often associated with certain infectious agents or mechanical tissue damage. A passive response to external injury, it is characterized by early loss of plasma membrane integrity, total cellular breakdown, and release of intracellular contents from the cell. The early breakdown of the plasma membrane in necrosis facilitates spilling of intracellular contents, which can contain immunostimulatory molecules, including heat shock protein Hsp10 [43]. In case of cancer cell death via necrosis, larger cfDNA fragments are observed than for apoptosis, exceeding 10,000 bp in human plasma [44] (Figure 1). Considering that necrotic processes in Parkinson’s disease predominantly affect the midbrain, only low concentrations of longer cfDNA fragments are expected to be present in the serum.

### 2.3. NETosis

NETosis is a process in which neutrophils release neutrophil extracellular traps (NETs) into the surrounding environment [45]. These NETs are web-like structures made up of chromatin, histones, and granule-associated proteins, and serve to trap and eliminate invading microorganisms such as bacteria, viruses, and fungi. The activation of NETosis involves a cascade of molecular events, including the generation of reactive oxygen species (ROS), the breakdown of the nuclear envelope, chromatin decondensation, and eventual NET release (Figure 1). A critical step in this process is the activation of the NADPH oxidase complex, which is triggered by an increase in intracellular calcium levels and leads to ROS production.

Mitochondria contribute to this process by producing ROS, which may influence NADPH oxidase activity and further promote NETosis [45]. NETosis is also an essential component of the innate immune response, enabling neutrophils to directly neutralize pathogens. This mechanism has also been investigated in the context of Alzheimer’s disease [46,47]. It was shown that neutrophils and NETs also accumulate around Aβ plaques in AD patients, whereas neutrophils from age-matched controls remain in the blood vessels. In another study by Smyth et al. (2022) [48], increased adhesion of neutrophils to small blood vessels, along with NETs, was observed in an AD mouse model and in AD patients, suggesting that NETosis contributes to BBB breakdown in AD, where evidence of NET formation and the release of cfDNA suggest a possible role for neutrophils and NETs in Parkinson’s neurodegeneration and tissue damage. The potential link to PD are still missing and might be worthwhile to pursue in the future. There is no evidence regarding the length of the cfDNA fragments resulting from NETosis.

### 2.4. Active Secretion

Another possible mechanism delivering cfDNA is active cellular secretion, involving exocytosis not only of cfDNA but also of proteins, ribonucleic acids (RNA), and lipids [49]. Studies indicate the length of cfDNA from active secretion to be approximately 2000 bp [50]. Active secretion allows living cells to actively release cfDNA [46,51,52]. Indeed, extracellular vesicles (EVs) are secreted by cells in both physiological and pathological conditions [46]. The mechanism of active secretion of cfDNA from a cell involves exosome and autophagy-dependent pathways, and it is difficult to differentiate its origin from passive release mechanisms. Although there is no evidence for detection of this mechanism in PD, recent studies on EVs present a promising PD therapy based on antisense oligonucleotide (ASO)-based gene therapy to reduce α-synuclein production [53].

## 3. Mitochondria Biology

**Figure 2:**
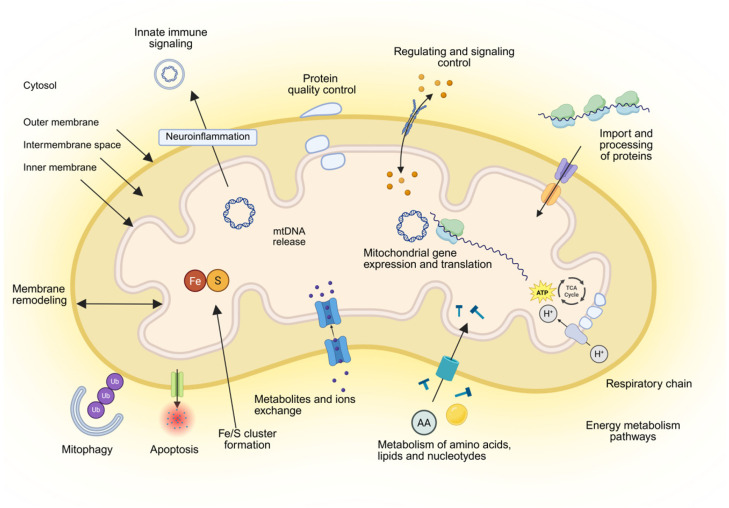
**Overview of mitochondria and their functions**. Mitochondria consist of four compartments: outer membrane, intermembrane space, inner membrane and matrix. Their main functions are energy metabolism composed of respiration and synthesis of ATP; metabolism of amino acids, lipids, and nucleotides; biosynthesis of iron-sulfur (Fe/S) clusters and cofactors; the import and processing of precursor proteins that are synthesized in the cytosol to express the mitochondrial genome; quality control and degradation processes, including mitophagy and apoptosis; signaling and redox processes; neuroinflammation, maintaining membrane architecture and dynamics; and mediating the tricarboxylic acid (TCA) cycle. cGAS and STING pathways. Created in BioRender. Ambrosius, F. (2025) https://biorender.com/4abae0m (accessed on 23 November 2025).

Mitochondria serve as the powerhouse of the cell, playing a central role in regulating numerous metabolic processes [54,55,56] (Figure 2). Mitochondria convert the energy stored in food into an electrochemical proton gradient across the inner membrane that drives mitochondrial ATP synthase, thus providing large amounts of ATP for the cell. In addition, mitochondria fulfill central functions in the metabolism of amino acids and lipids and in the biosynthesis of iron-sulfur clusters and heme [57]. Mitochondria engage in multiple cellular and extracellular signaling pathways by producing cellular ROS, important molecules involved in various redox-sensitive signaling pathways and mitochondrial nucleus crosstalk by regulation of gene expression.

Owing to their own genome, mtDNA located within the mitochondrial matrix, mitochondria can encode 13 proteins of the respiratory chain, 22 transfer RNA (tRNA), and 2 ribosomal RNA (rRNA). The genetic information of almost 1500 mitochondrial proteins required for the replication and expression of oxidative phosphorylation (OXPHOS) machinery is mostly stored in the cell nucleus [58]. Thus, over 90% of mitochondrial proteins have to be imported into their final mitochondrial destination through import machineries located within each mitochondrial sub-compartment [59,60,61,62]. These protein import machineries are regulated at multiple levels, including through cell cycle control [62]. Mitochondria have developed sophisticated communication pathways with other organelles through a tubular network of interactions with the endoplasmic reticulum (ER), facilitating regular fusion with other mitochondria or fission to separate and form new mitochondria [63,64]. Research on mitochondrial trafficking, primarily conducted in neurons, has yielded quantitative measurements of mitochondrial transport rates, which range from 0.39 ± 0.031 μm/min [31]. Mitochondrial fission protein 1 (FIS1) and dynamic-related protein (DRP1) control fission processes, whereas mitofusion proteins (MFN1/2) are regulators of mitochondrial fusion. The balance between fusion and fission, known as mitochondria dynamics, is crucial for cell homeostasis [65].

## 4. Mitochondria Dysfunction in Parkinson’s Disease

### 4.1. Mitochondrial Quality Control

The proper function of mitochondria is maintained through a cellular system known as mitochondrial quality control [66], which ensures the turnover of mitochondrial proteins and organelles. This system involves several pathways, including mitochondria-associated degradation, the ubiquitin-proteasome system, mitochondrial proteases (mitoproteases), and the selective removal of damaged mitochondria [67]. As previously described, mitochondria form a dynamic network that is continuously remodeled in fusion and fission processes. They are involved in the maintenance of cellular homeostasis and have been implicated in the pathogenesis of numerous neurodegenerative disorders such as Parkinson’s disease [68,69]. For over three decades, mitochondrial dysfunction has been associated with neurodegeneration in Parkinson’s disease; however, its precise role—whether as a trigger, driving factor, or secondary consequence—remains unclear. In Parkinson’s disease, mitochondrial dysfunction is evident through impaired complex I activity, reduced bioenergetic capacity, heightened oxidative stress, and diminished stress resilience. Mitochondrial impairment primarily arises from the inhibitory effects of aberrant α-synuclein and environmental toxins on complex I of the mitochondrial electron transport chain [70]. Notably, complex I deficiencies leading to ROS overproduction have been observed in brain tissue from individuals with sporadic PD. Furthermore, familial forms of early-onset PD, associated with mutations in the autosomal recessive genes PINK1 and Parkin, exhibit impaired mitochondrial selective degradation in the process of mitophagy. Elevated levels of mitochondrial DNA (mtDNA) deletions have been associated with respiratory chain deficiencies and selective neuronal loss in PD [71,72]. Furthermore, the discovery of genes such as PRKN, PINK1, DJ-1, and SNCA, which are linked to familial forms of PD, has highlighted a shared pathway involving mitochondrial quality control and dynamics (Table 1). The report of the identification of mitochondria-related gens by Zong et al., 2024 [73] revealed genes coding for the respiratory chain complexes were found to be downregulated in PD across all three datasets while genes related to apoptosis and immune response were significantly upregulated. Additionally, in study by Wang et al., 2025 [74] analyzed publicly available genome-wide association study (GWAS) summary statistics alongside data on 1136 mitochondria-related genes. From this analysis, they identified a subset of genes involved in mitochondrial function that showed significant associations with Parkinson’s disease (PD). Notably, increased expression of nuclear genes NDUFAF2, BCKDK, and MALSU1 was associated with a higher risk of developing PD. Additionally, increased somatic mtDNA mutagenesis leads to premature aging in mice, and mtDNA damage accumulates in the human brain with age and in PD. In the study by Dölle et al. (2016) [75], the full spectrum of mtDNA alterations—deletions, copy-number variation, and point mutations—was examined in single neurons from the dopaminergic substantia nigra and other brain regions of individuals with PD and neurologically healthy controls. It was shown that in dopaminergic substantia nigra neurons of healthy individuals, mtDNA copy number increases with age, preserving the pool of wild-type mtDNA despite the buildup of deletions. In PD, however, this compensatory upregulation fails, leading to a depletion of wild-type mtDNA. In contrast, the load of neuronal mtDNA point mutations is not elevated in PD. Interestingly, studies based on tissue homogenates, rather than individual neurons, have produced substantially contradictory results [76].

Nevertheless, these findings indicate that disrupted mtDNA homeostasis is a critical factor in the neuronal loss associated with Parkinson’s disease and carries significant implications for therapy-focused research. Pharmacologically increasing mtDNA levels could restore neuronal respiration and provide a neuroprotective effect in PD. The authors suggested treatment targeting peroxisome proliferator-activated receptor gamma (PPARγ), which is responsible for the mitochondrial biogenesis pathway and has been linked to a markedly reduced risk of PD, potentially compensating for somatic mtDNA damage [77].

### 4.2. Mitophagy

**Figure 3:**
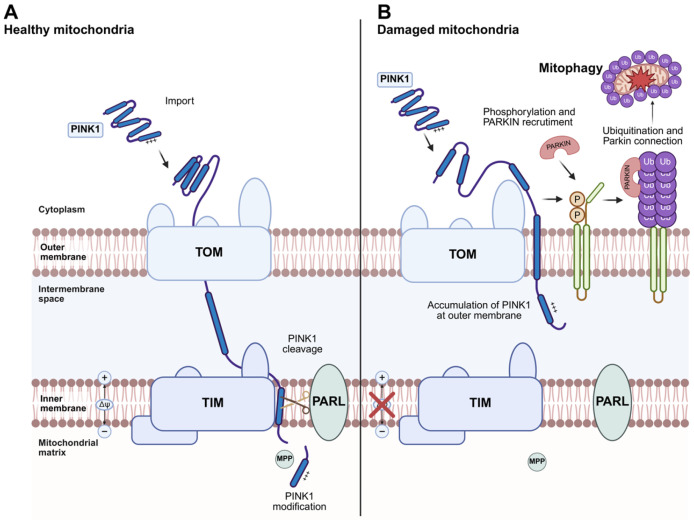
**PINK1/Parkin-dependent mitophagy.** (**A**) Under normal conditions, PINK1 is partially imported into mitochondria through the TOM (Translocase of the Outer Membrane) and TIM (Translocase of the Inner Membrane) complexes in a membrane potential (∆ψ)-dependent process. Within the inner membrane, the rhomboid protease PARL cleaves PINK1, promoting its partial degradation. (**B**) When mitochondria become damaged and lose their membrane potential (∆ψ), PINK1 import is blocked, leading to its accumulation at the TOM complex on the outer mitochondrial membrane. This accumulation recruits the E3 ubiquitin ligase Parkin to the mitochondria. PINK1 phosphorylates Mitofusin 2, which likely acts as a receptor for Parkin. Activated Parkin ubiquitinates outer mitochondrial membrane proteins, including mitofusins, marking the damaged mitochondria for degradation through mitophagy. Mutations in *PINK1* or *Parkin* have been identified in monogenic forms of Parkinson’s disease. Created in BioRender. Ambrosius, F. (2025) https://biorender.com/d2b1253 (accessed on 23 November 2025).

PINK1 and Parkin have been identified as central regulators of mitophagy, the processes responsible for removing damaged mitochondria [67]. This process requires the induction of general autophagy and the priming of damaged mitochondria mediated by the PINK1/Parkin signaling pathway. Mitophagy is important to maintain the quality of the mitochondrial pool and for regulation of mitochondrial abundance in response to environmental cues such as hypoxia [78]. During mitophagy, mitochondria are specifically sequestered into autophagosomes via receptor proteins that link mitochondria to the autophagy membrane [79] (Figure 3). Such autophagy receptors can either interact with ubiquitinated outer membrane (OMM) proteins or themselves be integrated into the OMM, but they have in common the ability to bind to ATG8 homolog proteins in the autophagy membrane via a specific LC3 interacting region (LIR) [80]. In mammals, mitophagy is generally divided into two main functionally distinct groups based on the requirement for the kinase PINK1 and the Ub E3 ligase Parkin, often referred to as PINK1/Parkin-dependent and PINK1/Parkin-independent mitophagy. The first pathway can be initiated while transmembrane potential is dissipated, while PINK1/Parkin-independent mitophagy does not require loss of the mitochondrial membrane potential [81].

Thus, the discovery that the familial PD genes PINK1 (PTEN-induced putative kinase 1) and parkin (PRKN) regulate mitochondrial degradation through mitophagy reinforced the significance of this pathway in PD pathogenesis. Recent advances have shed light on both the upstream and downstream regulators of canonical PINK1/parkin-dependent mitophagy, as well as on noncanonical mitophagy mechanisms activated by mitochondrial stress. Additionally, growing insights into the involvement of PD-associated genes such as SNCA, LRRK2, and CHCHD2 (Table 1) in mitochondrial dysfunction—and their intersections with sporadic PD (sPD)—are revealing new opportunities for mitochondrial-targeted therapeutic strategies.

### 4.3. PINK1/Parkin-Dependent Mitophagy

The mitochondrial kinase PTEN-induced putative kinase 1 (PINK1) and the mainly cytosolic Parkin, an E3 ubiquitin ligase, have been linked to the monogenic subtype of Parkinson’s disease. PINK1 and Parkin function as part of a mitochondrial quality control pathway that is likely impaired in Parkinson’s disease [82]. In healthy cells, PINK1 is partially imported into mitochondria via the mitochondrial membrane import translocases TOM (translocase of the outer membrane) and TIM23 complexes (translocase of the inner membrane) in a (∆Ψ)-dependent manner, and is then processed by the inner membrane rhomboid protease PARL (presenilin-associated rhomboid-like protein) (Figure 3A). PARL cleaves within the transmembrane segment of PINK1 and generates an amino terminus with a destabilizing amino acid residue. Cleaved PINK1 is then translocated to the cytosol, where the amino-terminal region is recognized by ubiquitin ligases, leading to degradation of PINK1 by the proteasome. When damaged mitochondria reveal dissipated transmembrane potential, PINK1 cannot be translocated to the inner membrane and is not processed. Thus, PINK1 accumulates at the outer membrane (Figure 3B) [83]. Outer-membrane-located PINK1 promotes the recruitment of Parkin to mitochondria, resulting in ubiquitination of several mitochondrial proteins. Thereafter, PINK1 phosphorylates ubiquitin chains to further stimulate the mitochondrial recruitment of Parkin and its E3 Ub-ligase activity. The generation of phosphorylated poly-Ub chains on OMM proteins leads to the recruitment of autophagy receptors that contain Ub-binding domains, such as OPTN and NDP52 (also known as CALCOCO2) [84], to the mitochondrial surface.

### 4.4. Mitochondrial-Derived Vesicles (MDVs)

MDVs comprise the next step of the quality control continuum, removing larger protein complexes and membrane proteins that may be refractive to proteolytic degradation within the mitochondria [85,86]. An example of cf-mtDNA as a cargo of MDVs was presented in a study on disease mutations in fumarate hydratase leading to an accumulation of fumarate. The elevation in fumarate led to the incorporation of mtDNA into MDVs, released from mitochondria to the cytosol where cGAS and STING pathways were activated, triggering innate immune signaling (in this review, the STING pathway are discussed in the next section). MDV biogenesis does not require the autophagy machinery, thus separating MDVs from selective mitophagy pathways [87]. In Parkinson’s disease (PD), brain-derived extracellular vesicles (EVs) are capable of traversing the blood–brain barrier, thereby safeguarding their molecular cargo from enzymatic degradation, and can be efficiently isolated from various biofluids. The molecular composition of EVs is strongly modulated by the specific pathophysiological state of the donor cell. Numerous microRNAs have been identified within EVs derived from PD patients across multiple biological matrices [86] however the study on DNA as a cargo of MDVs are missing.

An elegant study on MDVs purified from the serum of PD patients, analyzed by TEM and cryo-EM, revealed that these EVs exhibited a characteristic diameter of approximately 100 nm and displayed typical exosomal membrane structures [88]. In this study, mitochondrial proteins were detected as Tom 70—subunit of the TOM import complex, proteins of the OXPHOS complex but no DNA fragments were identified. Interestingly studies on macrophages are reported to fragment and release oxidized mtDNA via mitochondrial porin—VDAC (voltage dependent anion selective channel) [89] and not via MDVs.

## 5. Mitochondria and Neuroinflammation in PD

### Neuroinflammation

Cell death mechanisms provide a wide range of endogenous molecules evoking cell response, including nuclear DNA and RNA fragments, nucleotides and nucleosides, DNA-binding molecules, and temperature-shock proteins. These intracellular molecules have been named damage-associated molecular patterns (DAMPs) [90,91]. DAMPs normally reside inside the cell, playing diverse roles in homeostasis, but are released to the extracellular space when cells are exposed to stress. In addition, mitochondria harbor many DAMPs that can initiate a variety of inflammatory signaling pathways [92]. Besides cf-ntDNA, cf-mtDNA also acts as a DAMP [91,93,94].

**Figure 4:**
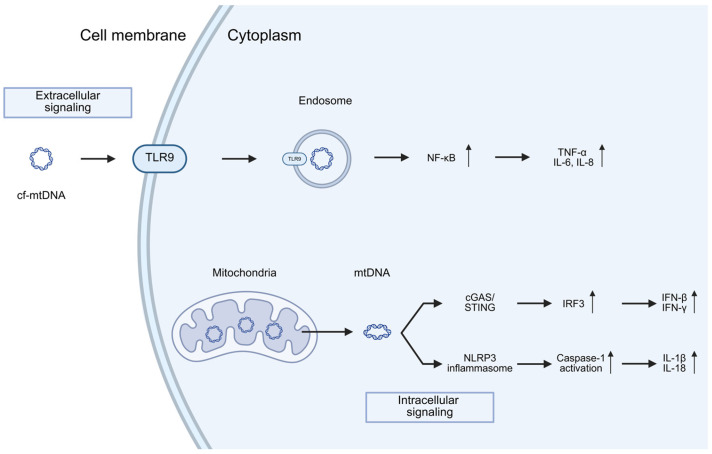
**Immune activation by mtDNA release through both intracellular and extracellular pathways.** TLR9 for sensing circulating (extracellular) mtDNA, and the NLRP3 inflammasome and the cGAS/STING pathway for sensing cytoplasmic (intracellular) mtDNA release. A host of downstream signaling pathways and cytokines are induced following activation of each of these distinct pathways (the details outlined in the text). Arrows up and down indicate up and down regulation. Created in BioRender. Ambrosius, F. (2025) https://biorender.com/eua44mm (accessed on 23 November 2025).

Interestingly, mtDNA is thought to trigger the innate immune response due to its bacterial ancestry and the presence of hypo-methylated CpG motifs [95]. In the process of neuroinflammation, mtDNA can instigate inflammation via interaction with pattern recognition receptors (PRRs), such as extracellular endosomal Toll-like receptors (TLRs) as TLR9—reported to recognize unmethylated CpG motifs from mtDNA or nucleotide-binding oligomerization domain (NOD)-like intracellular receptors (NLRs), or via the cyclic GMP/AMP (cGAMP) synthase (cGAS)/stimulator of interferon genes (STING) pathway [96,97,98] (Figure 4). cf-mtDNA activates cGAS to form a dimeric cGAS-DNA complex that synthesizes cyclic GMP-AMP (cGAMP) from ATP and GTP. This cGAMP functions as a second messenger because it is a high-affinity ligand for the ER membrane adaptor protein stimulator of interferon genes (STING). cGAMP induces conformational changes in STING, resulting in the subsequent activation of the transcription factors NF-κB and IRF3 through the kinases IKK and TBK1, respectively [92] (Figure 4). These responses culminate in the activation of interferon regulatory factors to enhance interferon secretion and interferon-stimulated gene expression. These PRR-expressing cells play a crucial role in the generation of neuroinflammation in neurodegenerative chronic diseases such as Parkinson’s (PD), Alzheimer’s disease (AD) and Huntington’s disease (HD) [99,100,101,102]. In the study by Jiang et al., 2023, the cGAS–STING signaling pathway activated by cf-mtDNA was stimulated in senescent PD astrocytes [103]. They found that mitochondrial DNA (mtDNA), but not nuclear DNA (nDNA), was obviously increased in both MPP+ and α-Syn PFF induced premature senescent astrocytes and long-term culture induced naturally senescent astrocytes by qPCR analysis. Notably, silencing astrocytic cGAS–STING signaling delayed both astrocyte senescence and Parkinson’s disease progression in MPTP-treated PD mice as well as in aged mice. Furthermore, they identified a downstream effector (LCN2) of the cGAS–STING pathway in the regulation of astrocyte senescence. Mechanistically, YY1 was found to act as a transcription factor that negatively regulates LCN2 expression. The nuclear translocation of YY1 was inhibited by its binding to STING. Collectively, these findings demonstrate that the cGAS–STING–YY1 axis promotes astrocyte senescence via upregulation of LCN2 expression, thereby contributing to the progression of Parkinson’s disease.

Additionally, cGAS, a cytosolic DNA sensor, becomes activated when it detects mtDNA within the cytoplasm what was also studied in PD cell model by Zhou et al., 2025 [104]. They observed a significant downregulation in the expression of molecules associated with mtDNA damage, namely PGC1α and TFAM, implying that mtDNA damage (oxidation) may trigger cGAS–STING–IRF7 signaling.

Remarkably, the genetic inactivation of STING has been shown to prevent inflammation, motor defects, and neurodegeneration in Parkin-deficient mice that had been subjected to mtDNA mutational stress, indicating a connection between mtDNA-induced inflammation and PD [105]. Interestingly, in the study by Tresse et al., 2023 it was found that the mechanism by which damaged mtDNA induces pathology related to PD in healthy neurons operates independently of cyclic GMP-AMP synthase (cGAS) and IFNβ/IFNAR signaling [106]. Instead, it involves the concurrent activation of TLR9 and TLR4 pathways, leading to increased oxidative stress and neuronal cell death, respectively. This study revealed singling pathway activated by endosome containing damaged mtDNA nor cf-mtDNA. Moreover the proteomic analysis of extracellular vesicles containing damaged mtDNA in PD revealed Ribosomal Protein S3—a protein reported to be involved in oxidative DNA repair [107] a TLR4 activator, as a critical mediator in the recognition and extrusion of damaged mtDNA. Authors suggested novel molecular pathways through which damaged mtDNA may initiate and propagate PD-like pathology, potentially offering novel opportunities for therapeutic intervention and disease monitoring. However, when damaged mtDNA is released into the extracellular space—either in a cell-free form or packaged within EVs—it can induce an infection-like pathology once taken up by healthy neurons (Figure 4).

## 6. Cell-Free DNA in Parkinson’s Disease

### 6.1. cfDNA in the Serum/Plasma of PD

Quantitative analysis using digital droplet PCR (ddPCR) demonstrated that serum levels of both cf-mtDNA and cf-nuDNA are significantly elevated in PD compared with healthy controls, with the increase in cf-mtDNA being markedly increased when compared to cf-ntDNA [108]. These results strongly implicate mitochondrial dysfunction and oxidative stress as contributing factors in PD pathology, likely reflecting ongoing neurodegeneration. In contrast, the study by Pyle et al. (2016) [109] reported reduced mtDNA levels in peripheral blood cells of PD patients. Importantly, their analysis was performed on mtDNA extracted from isolated blood cells rather than on cfDNA. Authors showed a significant reduction in mtDNA copy number in both peripheral blood and the substantia nigra pars compacta of PD patients relative to matched controls. This reduction reflects intracellular mtDNA depletion—most pronounced in affected brain tissue but also detectable in peripheral blood—and does not correspond to levels of cf-mtDNA. Therefore, this findings do not contradict those of Wojtkowska et al. (2024) [108]; instead, they describe decreased cellular mtDNA content, a parameter distinct from serum cf-mtDNA concentration. In accordance with a mouse model described by Borsche et al., 2020 (Table 2) [110], patients with biallelic or heterozygous PARK2/PINK1 mutations exhibited elevated serum levels of cf-mtDNA and IL-6 compared to either healthy control subjects or idiopathic PD patients (iPD). This indicates that cf-mtDNA levels offer predictive potential to discriminate between idiopathic PD and PD linked to heterozygous PARK2/PINK1 mutations. This could suggest different mechanisms of mtDNA dysfunction between idiopathic and genetic Parkinson’s disease. The study also confirmed that PD is associated with neuroinflammation due to the elevated IL-6 [111], as serum from PD patients is often enriched in pro-inflammatory cytokines, including TNF, IL-1b, IFNɣ, and IL-6 [112]. This is in line with a immunogenic function of mtDNA activating innate immune signaling pathways described in the review (Figure 4).

### 6.2. cfDNA Serum/Plasma Sequence Analysis in PD

Firstly, the newest study by Patil at al. (2025) concern NGS-based sequencing of serum-derived cfDNA from PD patients and identified of specific cfDNA molecules with differential levels in drug-naïve PD patients as compared to healthy controls. The obtained data were validated these cfDNA molecules in an independent drug-exposed PD (dopaminergic treatment) patient cohort. They have studied cf-ntDNA and showed its increase in both drug-naïve and drug-exposed PD serum, albeit with variations between the two groups, as compared to healthy controls [42] (Table 2). According to this study a total of 16 cfDNA regions showed differential levels in PD serum samples. BLAST and NCBI GenBank analyses indicated that none of the genomic or cfDNA regions exhibited a direct association with known genetic loci linked to Parkinson’s disease, neurodegeneration, or neuroprotection. Moreover the subsequent analysis showed that several cf-ntDNA regions mapped to both upstream and downstream areas of open reading frames [42]. The elevated levels concerns of 5 kb DNA regions across the genome in PD serum compared to controls including on chromosomes 2, 6, 8, 12, 17, and 19. Chromosomes 2, 8, and 12 showed multiple higher copy number regions while single regions were detected in chromosomes 12, 17, and 19. In case of heterogeneity of the PD the obtained results were validated from the drug-naïve PD cohort in an independent drug-exposed PD cohort and demonstrate potential utility of cf-ntDNA molecules as PD classifiers in both drug-naïve and drug- exposed PD patients [42]. Moreover this highlights that cf-ntDNA signatures may vary between these two main groups of patients. The identified chromosomes used for data analysis are from the human genome sequence (hg19—Genome Reference Consortium Human Build 37 (GRCh37) Homo sapiens genome assembly GRCh37—NCBI–NLM.

Secondly, in studies by Ying et al. in 2025 [113], analysis of the cfDNA telomer was performed on the plasma of PD patients, revealing that elevated levels of cf-ntDNA integrity, defined as the ratio of larger DNA fragments to total cf-ntDNA, can serve as an indicator of the relative contributions of different cell death mechanisms [114]. In this study it was compared four cf-ntDNA biomarkers regarding ALU sequence ALU: ALU115, ALU247. The cfDNA integrity (ALU247/ALU115), and cf-RTL (relative telomer length—telomere/ALU115). The levels of ALU115 and ALU247, which represent overall cf-ntDNA concentration and non-apoptotic cfDNA concentration, respectively, did not show significant differences when comparing the PD with the control group. In contrast, plasma cf-ntDNA integrity was significantly increased. Additionally, cf-ntDNA-RTL was significantly decreased in the PD compared with control groups. Interestingly the written study were also performed on the multiple sclerosis patients (MSA) and there were no significant differences found in ALU115; ALU247 and cfDNA integrity, and cf-ntDNA-RTL between the PD and MSA groups. According to the authors, the observed increased cf-ntDNA integrity in PD patients suggests a shift toward non-apoptotic cell death processes in neurodegeneration. Indeed, for PD, the loss of dopaminergic neurons in the substantia nigra reflects mostly necrosis, which typically results in the release of larger DNA fragments compared with apoptosis. These findings suggest that cf-ntDNA integrity and cf-ntDNA relative telomere length may serve as further promising biomarkers for the early diagnosis of Parkinson’s disease, potentially reflecting the specific underlying pathophysiological processes of these neurodegenerative disorders.

### 6.3. cfDNA in the CSF of PD Parkisnon’s Disease

In contrast, serum analysis of cfDNA from PD patients has revealed an opposite proportion of cf-mtDNA to cf-nDNA revealed cf-mtDNA levels in CSF are significantly decreased [108,109,115]. The authors suggested that reductions in cf-mtDNA are linked to the initiation, type, and duration of L-Dopa treatment. This study, performed on a large cohort of PD patients, also showed that cf-mtDNA levels correlate with comorbidities such as depression and insomnia; however, these associations were significant only when measured prior to treatment. The obtained results confirmed their previous study [116] conducted on a smaller group of PD patients using the same marker genes and qPCR technique (Table 2), although no correlation with cognitive impairment was observed.

**Table 2:** Summary of cfDNA associated with Parkinson’s disease.

Studies Sample/PD Subtypes	PD Subtypes	Types of cfDNA	Gene	Quantification Method	Methods of Analysis	Main Findings	References
10 HC 53 PD	iPD	mtDNA	*MTND1 MTND4* *B2M*	CSF	qPCR	Reduced copy number in PD patients compared to HC No correlation with cognitive impairment	[116]
10 HC 26 PD of EOPD	EOPD	ntDNA	N/A	CSF	methylation	2220 differentially methylated genes were identified; Aberrant methylation signatures were correlated with external factors	[117]
372 169 after treatment 250 114	PD	mtDNA	*MTND1* (minor deletion arc mitochondrial gene)	CSF	qPCR	ccf-mtDNA levels appear significantly reduced in PD cases when compared to matched controls and are associated with cognitive impairment; comorbidities and treatment can both influence ccf-mtDNA homeostasis,	[115]
262 HC 363 PD	PD	mtDNA	*MTND1 MTND4* *B2M*	PBC	qPCR	Decreased copy number in PD patients No correlation with cognitive impairment	[109]
17 HC 21 iPD 20 LRRK2 NMC * 26	iPD LRRK2-PD	mtDNA ntDNA	mt64-D1 mt96-D5 *TEFM-88* *TBP1–73*	CSF	ddPCR	Reduced copy number in PD patients compared to HC Higher proportion of mtDNA molecules with deletions in PD patients	[118]
57 HC 17 PD 55 HC 17 PD	mut+/+PD PRKN/PINK1 mut+/−PD PRKN/PINK1	mtDNA	*MT-ND1* *B2M*	serum serum	ddPCR ddPCR	cf-mtDNA is elevated in monogenic PD; results implicates inflammation due to impaired mitophagy and subsequent mtDNA release in the pathogenesis of monogenic PD	[110]
3HC 6PD	iPD	ntDNA		serum	NGS; qPCR	Increase in specific cfDNA molecules in both drug-naive and drug-exposed PD serum, albeit variations between the two groups, as compared to healthy controls	[42]
5 HC 13 PD	iPD	mtDNA	*COX*	CSF	ddPCR	Increased cf-mtDNA vs. cf-ntDNA	[108]
5 HC 13 PD	iPD	ntDNA	*KRAS*	CSF	ddPCR	Increased cf-ntDNA vs. control	[108]
15 HC 30 PD	iPD	mtDNA	*COX*	serum	ddPCR	Increased level of cf-mtDNA vs. cf-ntDNA in PD; Increased cf-mtDNA vs. control; increased cfDNA vs. control	[108]
15 HC 30 PD	iPD	ntDNA	*KRAS*	serum	ddPCR	Increased cf-ntDNA vs. control	[108]
72 HC 62 PD	iPD	ntDNA	*COX*	plasma	NGS	cell-free DNA integrity was significantly elevated whereas cell-free DNA relative telomere length was markedly shorter	[119]

Patients with Parkinson’s disease (PD)due to biallelic PRKN/PINK1mutations (mut+/+PD); affected heterozygous individuals (mut+/−PD); N/A not applicable; idiopathic Parkinson’s disease patients (iPD); non-manifesting carriers = NMC *.

### 6.4. cfDNA CSF Sequence Analysis in PD

Available data regarding cfDNA sequence analysis of CSF are limited. An analysis performed by Meng et al. (2021) on EOPD (early-onset Parkinson’s disease) identified 2220 differentially methylated genes in cf-nDNA, and clustering and enrichment analyses indicated aberrant neuronal function and immune responses [117]. Additionally, another study reported by Puigròs et al. (2022) [118] showed that cf-mtDNA can differentiate iPD from monogenic (LRRK2-associated) PD, and again confirmed the observation of reduced cf-mtDNA levels in PD.

### 6.5. Important Discrepancies of cfDNA Studies in PD

Several studies, primarily analyzing serum, plasma, and cerebrospinal fluid (CSF), performed a range of quantitative techniques, including quantitative PCR (qPCR), digital droplet PCR (ddPCR), next-generation sequencing (NGS), and methylation pattern analysis (Table 2). These studies vary in sample size, the mitochondrial and nuclear markers selected, and the Parkinson’s disease subtypes investigated (EOPD vs. iPD). Despite these methodological differences, the overall findings are largely consistent, although some discrepancies have been noted and are discussed above. Discrepancies in the obtained results concern the reciprocal levels of cf-mtDNA in serum versus CSF in PD compared to healthy controls. Interestingly, cf-mtDNA levels in CSF are decreased, which may seem paradoxical, as cell death is generally associated with the release of mtDNA, which would be expected to increase, rather than decrease, cf-mtDNA concentrations in PD [97]. In the early stages of mitochondrial loss, however, there may be a compensatory suppression of the normal baseline release of mtDNA. This may explain why low mtDNA levels, potentially serving as an early indicator of impending neuronal death, are detected during the initial phases of neurodegeneration in CSF [91]. The reduction in cf-mtDNA in CSF may also result from decreased mitochondrial copy number, which correlates with aging. In the study by Dölle et al. (2016), dopaminergic neurons of the substantia nigra in healthy individuals showed an age-related increase in mtDNA copy number, maintaining the pool of wild-type mtDNA despite accumulating deletions [75]. In Parkinson’s disease, this compensatory upregulation fails, leading to depletion of the wild-type mtDNA population, which may contribute to the reduced cf-mtDNA levels in CSF. Interestingly, Wojtkowska et al. (2024) observed higher levels of cf-mtDNA relative to cf-nDNA in healthy controls, which appears to support this hypothesis [108]. The data presented in Table 2 should be interpreted with caution, particularly for PBM serum cells (Pyle et al., 2016), where mtDNA was isolated rather than cf-mtDNA [109]. Therefore, the observed decrease in mtDNA copy number in PBM cells may reflect the effect of the mtDNA release from the cells. This observation aligns with studies in this review reporting increased cf-mtDNA levels in the serum of PD patients [75,108].

## 7. cf-mtDNA as Potential Biomarker of Parkinson’s Disease

Potential biomarkers for Parkinson’s disease (PD) have been reported in multiple body fluids—including cerebrospinal fluid (CSF), peripheral blood, saliva, and urine—as well as in tissues such as the brain, intestinal tract, and skin [120,121]. According to Zimmermann and Brockmann (2022) [120], research has primarily focused on inflammatory biofluid markers in blood and CSF from PD patient cohorts. Over 50 pro-inflammatory markers have been assessed in serum or plasma, but only seven markers (CRP, IL-1β, IL-2, IL-6, IL-8, IFN-γ, TNF-α) have been investigated in more than five studies. The most robust data are available for CRP [122,123,124], while findings for other markers are less consistent.

CSF studies are less common, with 26 pro-inflammatory markers assessed overall; however, only IL-1β, IL-6, IL-8, and TNF-α have been measured in four or more studies. Another group of biomarkers concerns genetically associated PD markers, such as LRRK2 (Leucine-rich repeat kinase 2), GBA (Glucocerebrosidase), and PRKN/PINK1 (Parkin/PINK1) [125,126,127]. Research on these genetic forms of PD is limited but generally shows inflammatory profiles similar to those observed in sporadic PD. Since Parkin and PINK1 are key components of the mitophagy pathway [128], their deficiency could lead to mtDNA release and activation of neuroinflammatory pathways (Figure 4).

In the study by Borsche et al. (2020) reported increased serum cf-mtDNA levels alongside elevated IL-6, a pro-inflammatory cytokine, in a large cohort of patients with monogenic or idiopathic PD [110]. The study also assessed CRP levels, showing that CRP increased with higher IL-6 concentrations in PRKN/PINK1 mutation carriers, as well as in idiopathic PD patients and healthy controls [129]. Notably, a positive correlation between IL-6 levels and disease duration was observed in affected PRKN/PINK1 mutation carriers but not in idiopathic PD patients, suggesting a specific effect of these genetic mutations on IL-6. Group differences in CRP levels further support IL-6 as a more specific inflammatory marker in monogenic PD.

Interestingly, L-dopa reportedly increases neuroinflammation in PD [130], and inflammation is associated with cf-mtDNA release [131]. Studies have investigated whether L-dopa treatment alters cf-mtDNA levels in PD patients or has no effect in L-dopa-resistant individuals. Lowes et al. (2020) [115] found that L-dopa is significantly inversely associated with cf-mtDNA levels, highlighting the need for further investigation. Multiple factors likely influence cf-mtDNA levels, including comorbidities [132,133]. Lowes et al. (2020) also reported that CSF cf-mtDNA levels may correlate with the onset of comorbidities such as cognitive impairment, anxiety/depression, and insomnia, but only in the absence of treatment [115]. This suggests that treatment effects in reducing cf-mtDNA may outweigh the impact of comorbidities on increasing it. These findings indicate a potential interplay between cf-mtDNA, PD medications, and treatments for comorbidities. Overall, cf-mtDNA levels appear to be influenced by treatment initiation, type, and duration, which somehow limits their utility as a biomarker for disease onset. Nevertheless, serum cf-mtDNA holds considerable translational potential as a biomarker of disease state and could help guide therapies aimed at modulating the innate immune response in Parkinson’s disease.

## 8. Perspective and Conclusions

Cell-free DNA can be considered a waste molecule in body fluids collected in pathological conditions or as an active molecule in physiological conditions. The particular subtype and distribution of cfDNA in blood might determine its activity. Limited studies on the serum and CSF of PD patients have shown that cf-mtDNA could be a promising target to study this neurodegenerative disease. In this review, we have shown that cfDNA is associated with Parkinson’s disease and mitochondria undoubtedly plays pivotal role in the distribution of cell-free DNA. Due to its bacterial-type mitochondrial DNA structure, it seems that cf-mtDNA presents higher resistance towards nuclease-dependent degradation compared to cf-ntDNA. Moreover, mtDNA was shown to be stable in CSF in neurological disorders, which could explain the higher cf-ntDNA occurrence in human serum, plasma, and CSF [70,75]. Although substantial progress has been made in uncovering the related mechanisms, much remains to be explored. Gaining a deeper understanding of DAMP release and its regulatory pathways could both support the development of new research tools and open avenues for innovative therapeutic strategies designed to reduce inflammation and tissue injury. Ultimately, such advancements may lead to improved outcomes in diseases characterized by excessive DAMP release. Further studies on the mechanisms that control cfDNA release and clearance are necessary to better elucidate its biological significance in Parkinson’s disease. Research on the relationship between mitochondrial quality control mechanisms and the release of cf-mtDNA is becoming important for understanding the role of mitochondria in Parkinson’s disease. Continued research stored within the cfDNA sequence may offer valuable insights into intracellular communication and reinforce its potential as a biomarker to advance the diagnosis and treatment of PD. Monitoring mitochondrial DNA levels may provide important information on disease progression and treatment efficacy, but further studies are needed to validate mtDNA as a reliable biomarker for this neurodegenerative disorder.

**Figure 5:**
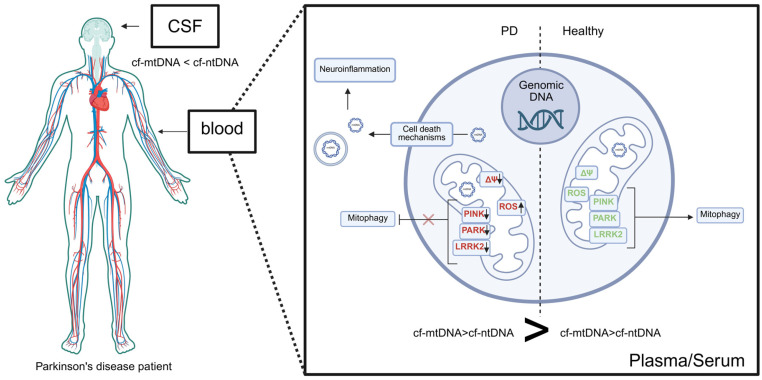
Summary picture. In Parkinson’s disease, cell-free mitochondrial DNA (cf-mtDNA) is released by cell death pathways as a result of oxidative stress, mitochondrial membrane depolarization and impaired mitophagy. Downregulation of key mitochondrial quality-control proteins (PINK1, PARKIN, LRRK2) further contributes to mitochondrial damage and promotes mtDNA release, which activates neuroinflammatory signaling (see text and Figure 4). mtDNA may be liberated either as cf-mtDNA or within endosomal structures. In the serum or plasma of PD patients, cf-mtDNA levels are elevated compared to cf-nDNA and healthy controls, whereas total cf-mtDNA in cerebrospinal fluid (CSF) is significantly reduced in PD patients relative to controls. This discrepancy may reflect differences in the sources and mechanisms of mtDNA release. Created in BioRender. Ambrosius, F. (2025). https://biorender.com/3uf7kvw (accessed on 23 November 2025).

In conclusion, the studies presented herein provide a comprehensive view of the relationship between mitochondria and cfDNA molecules in Parkinson’s disease. In the serum or plasma of PD patients, cf-mtDNA levels are elevated compared to cf-nDNA and healthy controls, whereas total cf-mtDNA in cerebrospinal fluid (CSF) is significantly reduced in PD patients relative to controls (Figure 5). This discrepancy may reflect differences in the sources and mechanisms of mtDNA release. In PD, cf-mtDNA appears to be primarily released through non-apoptotic cell death pathways, driven by oxidative stress, mitochondrial membrane depolarization, and impaired mitophagy. Downregulation of key mitochondrial quality-control proteins, including PINK1, PARKIN, and LRRK2 related to PD, further contributes to mitochondrial dysfunction and promotes mtDNA release, which can activate neuroinflammatory signaling pathways, such as IL-6-mediated responses (Figure 4). The reduction in cf-mtDNA in CSF may also result from decreased mitochondrial copy number, which normally increases with age to maintain the pool of wild-type mtDNA, as shown in dopaminergic neurons of healthy individuals; in PD, this compensatory mechanism fails, leading to depletion of wild-type mtDNA. Interestingly, studies on PBM cells indicate that decreased mtDNA copy number may reflect prior mtDNA release from cells, consistent with the observed increase in serum cf-mtDNA. Furthermore, serum cf-mtDNA may serve as a potential biomarker for PD, as it can discriminate between idiopathic PD and familial subtypes, including PD linked to heterozygous PARK2/PINK1 mutations and monogenic PD associated with LRRK2. We believe that a significant and promising strategy for improving diagnostic precision involves a multimodal, multi-analyte liquid biopsy approach that integrates cfDNA analysis with additional biomarkers, such as proteins (e.g., α-synuclein, neurofilaments, mitochondrial quality control proteins and other cytokines associated with neuroinflammation). Combining cfDNA and protein biomarker assessment can provide more detailed insights into the underlying disease pathomechanisms and substantially enhance the sensitivity and specificity of diagnostic procedures.

## Data Availability

No new data were created or analyzed in this study. Data sharing is not applicable to this article.

## Abbreviations

The following abbreviations are used in this manuscript:

PD	Parkinson’s disease
cfDNA	Cell-free DNA
cf-ntDNA	Nuclear derived cell-free DNA
cf-mtDNA	Mitochondrially derived cell-free DNA
CSF	Cerebrospinal fluid
LBs	Lewy bodies
SN	Substantia nigra
LRRK2	Leucine rich repeat kinase 2
CAD	Caspase-activated DNase
dsDNA	Double-stranded DNA
NETs	Neutrophil extracellular traps
ROS	Reactive oxygen species
AD	Alzheimer’s disease
RNA	Ribonucleic acids
ASO	Antisense oligonucleotide
EVs	Extracellular vesicle
DAMPs	Damage-Associated Molecular Patterns
tRNA	Transfer RNA
rRNA	Ribosomal RNA
ER	Endoplasmic reticulum
FIS1	Mitochondrial fission protein 1
DRP1	Dynamic-related protein 1
MFN1/2	Mitofusion proteins 1 and 2
LIR	LC3 interacting region
PINK1	PTEN-induced putative kinase 1
PTEN	Phosphatase and tensin homologue deleted on chromosome 10
TOM	Translocase of the outer membrane
TIM	Translocase of the inner membrane
PARL	Presenilin-associated rhomboid-like protein
OMM	Outer mitochondrial membrane
OPTN	Optineurin
NDP52	Nuclear dot protein 52
CALCOCO2	Calcium-binding and coiled-coil domain-containing protein 2
MDVs	Mitochondrially derived vesicles
cGAS	Cyclic GMP/AMP synthase
cGAMP	Cyclic guanosine monophosphate–adenosine monophosphate
STING	Stimulator of interferon genes
TCA	Tricarboxylic acid cycle
PRRs	Pattern recognition receptors
TLRs	Toll-like receptors
NOD	Nucleotide-binding oligomerization domain
NLRs	Nucleotide-binding oligomerization domain like receptors
HD	Huntington’s disease
ALS	Amyotrophic lateral sclerosis
MS	Multiple sclerosis
IRF3	Interferon regulatory factor 3
IKK	IkappaB kinase
TBK1	TANK-binding kinase 1
qPCR	Quantitatively polymerase chain reaction
PCR	Polymerase chain reaction
NGS	Next Generation Sequencing
TNF	Tumor necrosis factor
IFNɣ	Interferon gamma
IL-6	Interleukin 6
